# A New Perspective on the Role of Self-Confidence and Confidence in the Evaluation and Rehabilitation of Children With Adverse Life Experience and Borderline Intellectual Functioning: A Preliminary Study

**DOI:** 10.3389/fpsyg.2021.720219

**Published:** 2021-08-24

**Authors:** Annalisa Valle, Gisella Baglio, Michela Zanette, Davide Massaro, Francesca Baglio, Antonella Marchetti, Valeria Blasi

**Affiliations:** ^1^Research Unit on Theory of Mind, Department of Psychology, Università Cattolica del Sacro Cuore, Milan, Italy; ^2^IRCCS Don Carlo Gnocchi Foundation ONLUS, Milan, Italy

**Keywords:** borderline intellectual functioning, children, internal working model, attachment, adverse childhood experience, clinical treatment, rehabilitation

## Abstract

The borderline intellectual functioning (BIF) is a neurodevelopmental condition characterized by a borderline intelligence quotient (range 70–85) with difficulties in cognitive and social domains. Children with BIF often live in adverse conditions and show academic and behavioral difficulties. Rehabilitation programs for these children focus mainly on cognitive aspects, sometimes with the aid of new technologies that are able to engage and motivate. In this framework, the affective development of children with BIF and its possible role both in the difficulties they manifest and in the rehabilitation is still poorly investigated. In this work, we investigate the characteristics of the internal working models of these children by applying the separation anxiety test, using both the classical and a new coding system to identify the specific features of the attachment representation. Results delineate a profile characterized by low self-confidence and high separation anxiety, with a tendency to somatization. In the light of these results, we suggest that this attachment profile has an impact on the therapeutic relationships and on the efficacy in the use of technological devices. We propose a new perspective in which the interpersonal relationship with the psychologist and the support of the self-confidence of children are crucial to treating cognitive and behavioral difficulties in children with BIF. Only in this case, the use of new technologies and tools may be effective in promoting the greatest possible benefit from therapeutic interventions.

## Introduction

Adverse childhood experience (ACE) impacts neurological and psychological development lifelong (Hassiotis et al., 2019). It is widely known that experiences of threat and/or deprivation, such as unpredictable environment, lack of rules and routines, poor scaffolding, and significant communicative exchanges can affect attachment bonds and produce separation anxiety and externalizing disorders (Luecken et al., 2013). Moreover, the ACE impacts emotional processing and executive functioning with cascading effects on adaptive skills (McLaughlin, 2016).

A high-risk population to be exposed to ACE are children with borderline intellectual functioning (BIF), a condition in between typical development and intellectual disability (Hassiotis et al., 2019), characterized by heterogeneous profile including an intelligence quotient (IQ) within the range of 70–85, associated with difficulties in social participation and adaptability. The difficulties in cognitive and social functioning, specifically in executive functioning, language, learning, movement, emotion regulation, coping, and mentalization (Salvador-Carulla et al., 2013; Peltopuro et al., 2014; Hassiotis, 2015; Baglio et al., 2016; Contena and Taddei, 2017; Alesi et al., 2018; Predescu et al., 2020) expose individuals with BIF to the risk of developing mental illnesses lifelong (Hassiotis et al., 2019). Given these premises, it is crucial to find effective rehabilitation approaches for this population.

A growing body of literature explores the possibility of using technology such as virtual reality (VR), serious games, and applied games for the rehabilitation of neurodevelopmental disorders, with the purpose to intervene in several domains such as cognition, mental health, and social cognition. Specifically, VR, allowing for an ecological setting that mimicks real social interaction, has been widely used to train social cognition in children with autism spectrum disorder (Strickland, 1997; Wass and Porayska-Pomsta, 2014; Karami et al., 2021). Moreover, VR, applied games, and serious games have been extensively used to help children with mental health problems such as anxiety and depression (Halldorsson et al., 2021). Efficacy results are encouraging, although more studies are necessary. Indeed, despite good acceptance and adherence, in most cases, the effects of VR/games intervention were comparable with the effects of non-therapeutic games (Halldorsson et al., 2021). Finally, the need for a user-centered and personalized design to ensure that the needs and specificities of patients are more effectively met is well-recognized (Parsons et al., 2017; Halldorsson et al., 2021; Karami et al., 2021).

Accordingly, previous works (Baglio et al., 2020; Blasi et al., 2020b) investigated the effects of different rehabilitation programs for children with BIF, comparing interventions with and without the use of new technologies. Results highlighted how a game-therapy approach that applied highly engaging and motivating technological devices (Wii and Xbox) was not effective in promoting motor planning abilities in children with BIF, but, the use of a body-awareness approach which involved learning body management techniques, thanks to the relationship with the therapist, proved to be effective. The impossibility to personalize the treatment with the used technologies was considered the main factor explaining this result. In fact, the therapeutic relationship allows tailoring the rehabilitation programs to the specific characteristics of the subjects, taking into account emotional–relational factors that standard programs do not consider, even when using VR/serious games.

Typically, the rehabilitation programs for children with BIF focuses on executive functions, learning and language abilities deficits, neglecting the importance of personalizing these programs by adapting them to the emotional–relational context of the subjects and to their specific needs. The high risk of ACE of the children with BIF implies a lack of a responsive and mentalizing environment (Fonagy and Campbell, 2019) that exposes children to a high risk for insecure attachment and poor emotional processing and regulation (Cooke et al., 2019). The consequent emotional disruption can strongly affect learning and behavior. We argue that approaching the BIF difficulties considering self-confidence and attachment of children requires an intervention at the core of the problem. For instance, a secure base gives the possibility to explore the world, and this is at the base of any and each learning and social experience. Furthermore, aggressive and oppositional behaviors or withdrawal and introversion that often characterize children with BIF, can be more effectively dealt with if self-confidence and the type of attachment are considered.

In the present study, we propose a new perspective enhancing the role of self-confidence and attachment to understand the special needs of children with BIF and ACE and to tailor the rehabilitation intervention. To this purpose, we explore self-confidence and attachment in a small group of children with BIF and ACE through a descriptive analysis of their answers to the separation anxiety test (SAT) (Fonagy et al., 1997; Liverta Sempio et al., 2001), with the aim to explore their internal working model (IWM) characteristics.

## Materials and Methods

We recruited 22 children with BIF who attend mainstream primary school in Italy (age: *M* = 7.73; *SD* = 1.31; gender: M:F = 9:13).

Each child, for clinical/diagnostic purposes, underwent a clinical interview with both parents and teachers, and a neuropsychological assessment to evaluate the following: (1) the intellectual functioning with Wechsler intelligent scale for children IV (WISC-IV; Orsini et al., 2012). WISC-IV scores are expressed as standard scores with a mean of 100 and a standard deviation of 15; (2) the adjustment with Vineland adaptive behavior scale II (VABS-II; Sparrow et al., 2016), whose scores are expressed as standard scores with a mean of 100 and standard deviation of 15; (3) the presence of ACE with environmental stress check-list (ESCL; Blasi et al., 2020a), a check list comprising the V-code from DSM-V and Z-code from ICD-10 to evaluate environmental stress; and (4) the socio-economic status (SES) with four factors index (Hollingshead, 2011). The SES distinguishes five socio–economic classes: low (8–19), middle-low (20–29), middle (30–39), middle-high (40–54), and high (55–66).

Inclusion criteria are the presence of BIF (IQ range 70–85) and the presence of adaptive-behavior difficulties that could emerge either from Vineland's total score (bordeline score: 70–85) or from the score of the subscales, or from the clinical interview with parents and/or teachers.

Exclusion criteria were the presence of genetic or metabolic syndromes, major neuropsychiatric disorders (such as ADHD and autism spectrum disorder), and neurological conditions (epilepsy, traumatic brain injury, brain malformation, and infectious disease involving the central nervous system). A positive history for psychoactive drugs, particularly referring to current or past use of psychostimulants, neuroleptics, antidepressants, benzodiazepines, and antiepileptic drugs was also considered exclusion criteria.

To investigate the IWM, all children were tested with the SAT.

### Separation Anxiety Test

The SAT assesses the IWM of the children referred to the relationship with their parents. It is a semiprojective test composed of six items, each represented by a picture introduced by a short storytelling about the separation between the mother (or mother and father) and the daughter/son. To facilitate the identification of the child with the character in the picture, the latter is called with the same name. Three items describe separations of “high intensity” and three items describe separations of “low intensity,” depending on how long the mother was away from the child (for example, a separation of one morning in the first case and a separation of 2 weeks in the second case). At the end of each story, the psychologist asks the participant three questions related to the following: emotion, justification, and coping. The “emotion” question asks the child to identify the emotion of the character; children with secure attachment usually attribute a negative emotion to the character in the high-intensity items and a positive or mixed emotion in the low-intensity items. The “justification” question is with regards to the detection of the origin of this emotion. The justification of the children can be consistent with the emotion declared, showing a coherence of thought typical of secure attachment, or children can justify the emotion in a confused way, internally incoherent and/or not consistent with the situation, highlighting a difficulty to face the separation typical of the insecure attachment. The interpretation of the “emotion” and “justification” questions gives insight relative to the presence of avoidance. Finally, the “coping” question evaluates the capacity of children to anticipate what she/he would have done to cope with the situation; it represents a cue about the ability of the child to reflect on stressful situations and her/his tendency to deal with the separation. The coding system evaluates all the answers of the children for each item together; the three items proposing low-intensity separations receive a score on the self-confidence scale (SAT-SC; score 1–4), the three items proposing high-intensity separations receive a score on the attachment scale (SAT-AT; score 1–4), and all items receive a score on the avoidance scale (SAT-AV; score 1–3). Combining these scores, each child receives a final total score (SAT-TOT) in the range of 6–36, indicating the level of security in the attachment representation, according to which higher scores correspond to a more secure IWM.

To better appreciate the specific characteristics of the IWM of each child, a descriptive analysis of each question of the SAT was performed. First, we categorized the answer to the “emotion” question into positive, negative, and mixed. Secondly, we evaluated the “justification” answers. In the low-intensity items they were then evaluated along the self-confidence scale and organized into two categories, high (score 3 or 4) and low (score 1 or 2) self-confidence; in the high-intensity items, these answers were evaluated along the attachment scale, dividing the score into the same two categories high and low. All “justification” answers were also evaluated along the avoidance scale into the same two categories, high (score 2 or 3) and low (score 1) avoidance. Moreover, we added the somatization category, counting the answers that referred to a bodily reaction, such as having stomach-ache, as an emotion or a justification. The last step involved the “coping” question. According to the categorization proposed by Ann Easterbrooks and Abeles (2000), we classified the coping answers as “low” and “high.” The low category included non-active coping strategies (the child does not take specific actions to deal with the situation), punitive behavior, behavior that increases the distance between the child and the caregiver and non-answers; the high category included the use of multiple strategies, the research of social support, and positive and adaptive behavior. To summarize, we categorized each low-intensity item as high or low on the self-confidence, avoidance, and coping scales, and for each high-intensity item, we categorized as high or low the attachment, avoidance, and coping scales. Moreover, for each item, the number of answers related to somatizations was calculated and the type of emotion was classified.

Two independent coders (AV, and GB) coded the SAT separately. For each discording item agreement was reached upon discussion, and in case it was needed a third evaluator (VB) intervened.

## Results

Demographic characteristics and scores at neuropsychological scales included in the evaluations are illustrated in Table 1. Children showed an SES falling into the range from low to middle and were exposed to one or multiple environmental stressors (ESCL scoring). Global intellectual functioning was borderline, according to the inclusion criteria, and adaptive functioning ranged from borderline to normal. Moreover, attachment assessed with the SAT indicated an intermediate level of security (mean score = 25.91; s.d. = 3.89) at the border with insecure attachment (scores ≤ 24).

**Table 1:** Demographic characteristics and neuropsychological scores.

**Variables**	***N***
Sample size	22
Gender M:F	9:13
	**Mean (Sd)**
Age	7.73 (1.31)
SES	25.22 (11.06)
ESCL	4.82 (3.59)
VABS II_total scale	83.80 (9.69)
IQ_WISC IV	75.77 (7.52)
SAT_total scale	25.91 (3.89)

Table 2 shows the results of the descriptive analysis of the answers at SAT. We note that in the high-intensity items children showed a high level of attachment security because they recognized negative emotions and were able to justify them in relation to the separation from the mother; moreover, a low-avoidance score was observed. At the same time, in the low-intensity items participants recognized emotions mostly negative, associated with a low-self-confidence and avoidance scores.

**Table 2:** Classification of the answers to the “emotion,” “justification,” and “coping” questions of the SAT.

**SAT item**		**Quality of emotion %**			**Justification of emotions %**						**Coping %**		**SOM. %**
					**Attachment**	**Attachment**	**Self-Confidence**	**Self-Confidence**	**Avoidance**	**Avoidance**		
		**Positive**	**Negative**	**Mixed**	**Low**	**High**	**Low**	**High**	**Low**	**High**	**Low**	**High**	
Low intensity	1	22.72	77.27	0	NA	NA	77.27	22.72	54.54	45.45	95.45	0	31.81
	3	45.45	50	4.54	NA	NA	59.09	40.91	68.18	31.81	59.09	40.91	0
	4	22.73	77.27	0	NA	NA	90.91	9.09	86.36	13.64	50	50	4.54
	Examples	(3) Happy	(4) Sad	(1) A bit sad and a bit happy	NA	NA	(4) He doesn't move away because he can't hear what his parents are saying	(4) She wants to play with her friends	(1) He is tired and sleeps	(3) I don't know what it will do	(1) He will stay in bed all day	(3) He will study and then play at halftime with friends	She has a cold
	Mean score %	30.18	68.18	1.51	NA	NA	75.76	24.24	69.69	30.3	68.18	30.30	12.12
High intensity	2	4.54	95.45	0	31.84	68.18	NA	NA	72.73	27.27	72.73	27.27	4.54
	5	13.64	86.36	0	9.09	90.91	NA	NA	95.45	4.54	59.09	40.91	0
	6	29.09	81.82	29.09	27.27	72.73	NA	NA	81.82	18.18	54.54	45.45	0
	Examples	(2) She feels good	(6) Very sad	(5) A bit sad and a bit happy	(5) She doesn't want to stay with the babysitter	(6) He misses his mother	NA	NA	(6) He has not seen his parents for too long	(5) He plays many games with the babysitter	(2) He will wait for mom on the sofa	(6) He will call mom on the phone and ask her to come back	(2) She has fever
	Mean score %	15.76	87.88	9.70	22.73	77.23	NA	NA	83.33	37.88	62.12	37.88	1.51

In more detail, in the “emotion” question, the children with BIF responded in most cases with negative emotions both in the high (item 2: 95.45%, item 5: 86.36%; item 6: 81.82%) and in the low (item 1: 77.27%; item 3: 50%; item 4: 77.27%) intensity items. Regarding the “justification” question, in the low-intensity items, most parts of the answers belonged to low self-confidence (item 1: 77.27%; item 3: 50.09%; item 4: 90.91%); this indicates the difficulty of the children in the organization of thinking about separation, that is, based on confidence in the ability of an individual to deal with a low-stressful separation. In the high-intensity items, the scores of the attachment scale are high (item 2: 68.18%; item 5: 90.91%; item 6: 72.73%) and the scores in the avoidance scale are low (item 2: 72.73%; item 5: 95.45%; item 6:81.82%). Moreover, we individuated a percentage of somatization answers, specifically in the low-intensity item 1 (31.81%) and, on a smaller scale, in the item 2 (4.54%), and in the high-intensity item 4 (4.54%). Regarding the “coping” question, the first two items showed the highest percentage of low-coping answers (item 1: 95.45%; item 2: 72.73), characterized by poor and non-active strategies, whereas in the others a more balanced percentage of high and low coping was found, with a prevalence of low coping.

## Discussion

In this work, we proposed a description of the answers to the SAT of children with BIF, with the aim to explore their IWM characteristics.

First result refers to the total score of the SAT. The Italian validation of the test proposes three classes of scores, indicating, respectively an IWM secure, intermediate, and insecure. The children with BIF obtained a mean score of 25, the minimum score for the intermediate attachment at the border of the insecure attachment.

The specific descriptive analysis of the answers to each item provides useful indications to better understand the results. In children with BIF, the low-total score depended on a specific pattern of answers. Both in the case of high and low-intensity items, the answers evidenced intense negative emotions. If for the high-intensity items this pattern is typical of the secure attachment, in the low-intensity items these answers indicate an insecure IWM. In fact, children with a secure bond experienced and internalized the possibility to be separated from the caregiver for a limited period; consequently, they do not feel negative emotions, but they are able to live positively during this separation because they have confidence in their ability to cope with the situation. In the case of children with BIF the answers of low-intensity items are characterized by negative emotions, low-self-confidence justifications, and non-active coping strategies. This seems to indicate that for these children, all types of separations generate a high level of anxiety and that they did not develop confidence in their ability to manage these situations. A similar pattern has been previously reported in children with disabilities, e.g., intellectual disabilities (Green et al., 2015). From a clinical point of view, these data are of great interest because of their implications in all relational life contexts, such as school, family, and friendship; consequently, they exert relevant effects on learning and socialization, and in the rehabilitation context. With respect to rehabilitation contexts, it is a widely accepted assumption that new technologies are very effective in motivating and engaging children and allow for more ecological settings. Specifically, VR/serious games have also been effectively used in complex and difficult relational and behavioral contexts such as autism spectrum disorders. Nevertheless, the use of new technologies does not replace the importance of personalized pathways that consider the specific characteristics of young patients and their environment, and this may explain the limited efficacy observed in some cases (Halldorsson et al., 2021; Karami et al., 2021). Our experience, for example, tells us that whatever the approach, the relational aspect remains crucial for the effectiveness of rehabilitation intervention. Previous studies from our group investigated different rehabilitation approaches with and without the use of technological devices aimed at improving cognitive, affective, and motor abilities domains in children with BIF. Specifically, we used a multimodal approach named movement cognitive and narration of the emotions treatment (MCNT), in which the relational dimension was the core of the treatment. Results of our studies showed that despite the positive and motivating properties of the technology involved, the relational dimension was decisive in promoting significant results in all three domains in this population (Baglio et al., 2020; Blasi et al., 2020b).

In this work, we individuated self-confidence as a crucial relational dimension that could play a role in the rehabilitation treatment. Self-confidence is at the base of the sense of agency and self-efficacy, both aspects involved in the motivational attitude and in the perceived ability to manage different situations. Moreover, low self-confidence, as a structural aspect of the IWM, reflects a low confidence also in the others. Based on the Bowlby hypothesis (1973), Sroufe (2005) argues that the individual level of self-confidence is built firstly into the relationship with the caregivers (mainly the mother); infants who can consider their mothers as a secure base develop a high level of self-confidence, whereas infants in insecure attachment bonds will become children with low self-confidence, emotionally dependent on the adults in daily life. The self-confidence in the IWM becomes in time a relational model applied to all new relationships, and to have low confidence in the primary caregiver and in themselves implies the tendency to have low confidence in others, such as teachers, therapists, and peers (Rotenberg et al., 2013). This has important consequences in multiple life-contexts of the children. At school, for example, children with BIF who experience negative emotions in their relationship with the teacher and who have no confidence in their capacity to cope with the separation anxiety could activate themselves in an “alarm mode” (Bak, 2012; Valle et al., 2016, 2018), a defensive modality aimed to maintain closeness and affectivity with the teacher to obtain protection and care. This attitude limits exploration, participation, and learning from both academic and social perspectives. Also in the clinical setting, to feel negative emotions and to not have confidence in the ability of an individual to manage the separation from the therapist could have crucial consequences. The therapeutic intervention is based on the relationship, in which to have confidence in the other and in themselves is essential to cope and accept the intrinsic frustration. The oppositional behavior frequently observed in children with BIF could indeed be a consequence of their poor self-confidence and it manifests when the affective-relational dimension is not considered.

The characteristics of the IWM of children with BIF can be explained by referring to the ACE they live and to their specific cognitive deficit. Regards the first aspect, several ACE conditions such as parental conflict, substance abuse, or mental illness are associated with the failure of the parents to respond adequately to the needs of the child, and to be sensitive and responsive to the child. Consequently, these children are at risk to construct attachment bonds less secure than children in typical situations (McLaughlin, 2016). Regards the second aspect, the “dynamic maturational model of attachment” (Crittenden and Dallos, 2009) suggests that the representation of the affective experiences is a result of three-information processing processes referred to the somatic, cognitive, and affective domain. We assume that the deficit in the cognitive and social/affective information processing characteristic of the children with BIF determines a difficulty to elaborate the information about the separation, always considered dangerous and emotionally intense, which in turn implies the low justification in the SAT. Moreover, this can explain the presence of somatization answers because we can assume that children with BIF struggle to incorporate their own bodily sensations generated by the separation in this complex elaboration process, and then these sensations emerge in the analysis of the separation situations of children in the SAT.

The perspective we proposed represents a preliminary approach to the topic due to the small sample included and to the lack of a control group. In fact, the presence of a control group could provide further information regarding not only the characteristics of the IWM of the BIF children, but also their specific features compared with typically developing children. To support our perspective, future research studies with a larger sample and adopting possibly a longitudinal approach are needed. Nevertheless, we believe that the results discussed here shed new light on the importance of focusing, in the rehabilitation process, not only on the cognitive skills of children with BIF (as many therapeutic approaches already propose), but primarily on the affective aspects such as self-confidence. In our opinion, the use of new technologies (Di Dio et al., 2020) can be very useful to motivate and engage children and implement specific skills, but can be effective only if applied as a tool to support the therapeutic relationship and not as a substitute of the latter. When deficient, the confidence of children can be built in this relationship through an internalization process. This is a fundamental aspect that impacts all other skills that these children may develop and use in multiple life-contexts.

## Data Availability Statement

The raw data supporting the conclusions of this article will be made available by the authors, without undue reservation.

## Ethics Statement

The studies involving human participants were reviewed and approved by Ethics Committees of the Don Gnocchi Foundation, protocol number 2_18/02/2015. Written informed consent to participate in this study was provided by the participants' legal guardian/next of kin.

## Author Contributions

AV: conceptualization, methodology, and writing. GB: conceptualization, investigation, and writing. MZ: conceptualization and methodology. DM: conceptualization. FB: conceptualization and project administration. AM: supervision. VB: project administration, conceptualization, writing, and supervision. All authors contributed to the article and approved the submitted version.

## Conflict of Interest

The authors declare that the research was conducted in the absence of any commercial or financial relationships that could be construed as a potential conflict of interest.

## Publisher's Note

All claims expressed in this article are solely those of the authors and do not necessarily represent those of their affiliated organizations, or those of the publisher, the editors and the reviewers. Any product that may be evaluated in this article, or claim that may be made by its manufacturer, is not guaranteed or endorsed by the publisher.
